# Association between dietary theobromine with depression: a population-based study

**DOI:** 10.1186/s12888-022-04415-y

**Published:** 2022-12-06

**Authors:** Xin-yu Li, Hui Liu, Lu-yu Zhang, Xi-tao Yang

**Affiliations:** 1 Department of Interventional Therapy, Multidisciplinary Team of Vascular Anomalies, Shanghai Ninth People’s Hospital, Shanghai Jiao Tong University, No.639 Zhizaoju Road, Huangpu District, Shanghai, 200011 People’s Republic of China; 2 Department of Neurosurgery, Shanghai Ninth People’s Hospital, Shanghai Jiao Tong University, Shanghai, People’s Republic of China; 3 Department of Nephrology, Shanghai Jiao Tong University Affiliated Sixth People’s Hospital, Shanghai, China; 4 The Department of Kidney Transplantation, The First Affiliated Hospital of Zhengzhou University, Zhengzhou, China

**Keywords:** Depression, Theobromine, NHANES

## Abstract

**Objective:**

The purpose of this study is to investigate the possible link between dietary theobromine intake and symptoms of depression.

**Materials and methods:**

These results are based on the responses of 3637 people who took part in the National Health and Nutrition Examination Survey in 2017–2018. Participants' daily theobromine intake was determined using a 24-h food questionnaire from the 2017–2018 cycle. Presence of depression was defined as a score of 5 or above on the Patient Health Questionnaire. Association between theobromine intake and depression was examined using a multivariate logistic regression adjusting for several relevant sociodemographic, lifestyle and health-related factors.

**Results:**

A total of 6903 participants were included in the study. The results of multivariate logistic regression showed a correlation between depressive symptoms and theobromine intake (OR:1.17, 95%CI:1.02–1.34).

**Conclusions:**

Our cross-sectional population based study suggests that increased theobromine intake is associated with increased risk for depression. Nevertheless, more investigations are needed to confirm our findings.

## Introduction

Depression is a serious condition affecting 246 millions of individuals worldwide [1]. Depression is the most common cause of disability and the fourth most common contribution to the overall illness burden in the world [2]. Antidepressant drugs, psychotherapies, and a variety of brain stimulation methods are all validated therapy options for depression [3]. Antidepressants are one of the most often recommended groups of psychotropic drugs for adolescents in the United States [4]. However, patient adherence was quite poor, with as many as half of patients interrupting their therapy in the first six weeks [5]. Increasing data also shows that dietary factors have an impact on depression symptoms [6, 7]. Previous studies have reported a protective effect of chocolate against depression [5].  In this regard, one of the primary ingredient of chocolate, theobromine, has been show to protect cognitive function by regulating neurotransmitter signaling [8]. Despite this, few population-based studies have investigated the link between theobromine in the diet and depression. Therefore, we aimed at examining the association between theobromine consumption and depressive symptoms taking advantage of a large population-based cohort in the United States.

## Materials and methods

### Study population

The National Health and Nutrition Examination Survey, also known as NHANES, is an ongoing survey that is carried out on a rolling basis in order to collect cross-sectional data from the civilian population in the United States that does not reside in institutions [9]. Since 1999, the NHANES has been conducting a survey of a nationally representative, complicated, stratified, multi-stage probability sample of the US population [10]. Each wave of the survey has included a different participant. The assessment procedures include a household interview and a physical examination at a mobile examination center (MEC) [11]. In this study, we obtained data from 2017–2018. In NHANES, depressive symptoms were only assessed in patients aged 18 years; therefore, we only included data from this age group.

### Measures

#### Exposure: Theobromine intake

Participants in the NHANES were asked to take part in an in-person household interview as well as a health examination at a MEC, which included a recall of their dietary intake over the previous 24 h [12]. The Automated Multiple Pass Method, which was utilized in NHANES in order to collect dietary data, has been successfully validated [13]. More details are available at www.ars.usda.gov/ba/bhnrc/fsrg. Based on the distribution of theobromine intake in NHANES, we defined increased theobromine intake as values above the third quartile (Q3), ie 43 mg/day [8].

#### Outcome: depressive symptoms

The Patient Health Questionnaire (PHQ-9) is a validated 9-item depression screener that was used to evaluate depressive symptoms. The questions on this screener enquire about the duration and severity of depressive symptoms during the last two weeks [14]. For each question, the score ranged from 0 to 3, and the total score ranged from 0 to 27. Depressive symptoms were then categorised as "none or minimal" (0–4), "mild" (5–9), "moderate" (10–14), "moderately severe" (15–19), or "severe" (20–27) [15]. Depressive symptoms were defined as a score of ≥ 5 on the PHQ-9 [8].

### Covariates

Age, gender, race (Mexican American; white; black and other), multimorbidity, education level (below high school; high school and college or above), smoking status (former; never and current), drinking status (never; former; light; moderate and heavy) and the poverty income ratio (PIR) were all taken into consideration when determining socio-demographic characteristics. A never smoker is an adult who has never smoked or has smoked fewer than 100 cigarettes in their lifetime; former smokers are individuals who have reported smoking 100 cigarettes in their lifetime but are not currently smokers; and current smokers are individuals who have smoked 100 cigarettes on some days or every day in their lifetime [16]. Never drinkers reported consuming less than 12 drinks; ever drinkers reported having more than 12 drinks in their lives but not in the preceding year; and current drinkers were further categorized as light, moderate, or heavy current drinkers. Heavy current drinkers were defined as women drinking 3 drinks per day and men drinking 4 drinks per day, with 5 or more binge drinking days per month; moderate drinkers were classified as women drinking 2 drinks per day and men drinking 3 drinks per day, with 2 binge drinking days per month. People who drank just a little: did not satisfy the standards outlined above [17]. As a measure of socioeconomic status, the PIR, which is the ratio of total family income to the poverty threshold, was used: low (PIR < 1.35), medium (1.35 ≤ PIR < 3.0), and high (PIR ≥ 3.0) [18]. The presence of diabetes mellitus was defined as the need for the administration of insulin or oral antidiabetic medication treatment. Prediabetes is defined in this study by impaired fasting glucose and impaired glucose tolerance [19]. Body mass index (BMI) was determined by dividing weight in kilograms by the square of height in meters and was classified as underweight (BMI = 18.5), normal weight (BMI = 18.5—24.9), overweight (BMI = 25—29.9), and obese (BMI > 30.0) were the four BMI categories. Multimorbidity, defined as the presence of two or more chronic conditions in a person, has been linked to depression [20, 21]. Cardiovascular disease (CVD), chronic obstructive pulmonary disease (COPD), chronic kidney disease (CKD), asthma, arthritis, cancer, stroke, hypertension, hyperlipidemia, diabetes, and obesity were chosen based on their clinical importance and the availability in NHANES [20]. We used a backward stepwise regression to identify our final model with depression ( ie > 5 on the PHQ9) as our dependent variable. Improvement in the model was assessed using the Akaike Information Criteria (AIC). Two variables, [PIR, education], were excluded due to high collinearity.

### Statistical analyses

All statistical analyses were performed with R, version 4.0.5 (R Project for Statistical Computing) using the survey package, version 4 1–1 and with Free Software Foundation statistics software, version 1.3. In all tests, *P* < 0.05 (2-sided) was considered to indicate statistical significance. The categorical variables were summarized as percent and frequency, while continuous variables were summarized as mean and 95% confidence intervals (CIs). Categorical data were compared using the χ2 test or Fisher's exact test, while continuous data were compared using Student's t-test. The analyses were restricted to participants who had complete data records.

## Results

### Population characteristics

**Fig. 1:**
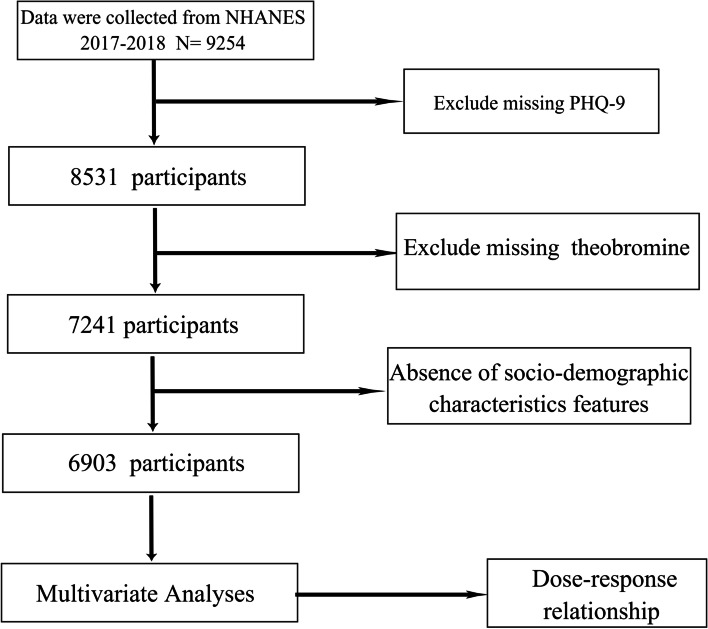
Flowchart of the study population

Figure 1 depicts the recruitment and inclusion/exclusion criteria for the study. The study included 6903 representative U.S. participants. Participants with depressive symptoms were younger than those without symptoms (49.0 ± 18.3 years versus 51.7 ± 18.0 years years, *P* < 0.05) (Table 1). Moreover, participants with increased theobromine intake (> = 43 mg/day) reported more depressive symptoms (34.9% vs 31.3% for low intake group, *P* < 0.05). In addition, the proportion of patients with depressive symptoms was higher in women and individuals with white ethnicity, lower family income, college or higher education, as well as multimorbidity. Similar differences were noted in never smokers and mild alcohol users.

**Table 1:** Characteristics of the overall target population according to theobromine

Variables	Total (*n* = 6903)	No depression (*n* = 5148)	Depression (*n* = 1755)	p
Theobromine intake, n (%)				0.005
< 43 mg/day	4677 (67.8)	3535 (68.7)	1142 (65.1)	
> = 43 mg/day	2226 (32.2)	1613 (31.3)	613 (34.9)	
Age, Mean ± SD	51.0 ± 18.1	51.7 ± 18.0	49.0 ± 18.3	< 0.001
Sex, n (%)				< 0.001
Female	3563 (51.6)	2543 (49.4)	1020 (58.1)	
Male	3340 (48.4)	2605 (50.6)	735 (41.9)	
Race, n (%)				< 0.001
Black	1135 (16.4)	895 (17.4)	240 (13.7)	
Mexican American	535 ( 7.8)	414 (8)	121 (6.9)	
Other Race	1814 (26.3)	1461 (28.4)	353 (20.1)	
White	3419 (49.5)	2378 (46.2)	1041 (59.3)	
PIR, n (%)				< 0.001
Low	1497 (24.3)	899 (19.7)	598 (37.4)	
Medium	2021 (32.9)	1381 (30.3)	640 (40.1)	
High	2634 (42.8)	2275 (49.9)	359 (22.5)	
Education, n (%)				< 0.001
Below high school	824 (11.9)	560 (10.9)	264 (15.1)	
High school	1593 (23.1)	1070 (20.8)	523 (29.8)	
College or above	4480 (65.0)	3513 (68.3)	967 (55.1)	
Smoke, n (%)				< 0.001
Former	1570 (22.7)	1126 (21.9)	444 (25.3)	
Never	4077 (59.1)	3278 (63.7)	799 (45.5)	
Now	1256 (18.2)	744 (14.5)	512 (29.2)	
Alcohol user, n (%)				< 0.001
Heavy	1038 (19.3)	672 (16.5)	366 (28)	
Mild	2565 (47.6)	1994 (48.8)	571 (43.7)	
Moderate	1005 (18.6)	748 (18.3)	257 (19.7)	
Never	783 (14.5)	671 (16.4)	112 (8.6)	
Multimorbidity, n (%)				< 0.001
No	2204 (31.9)	1702 (33.1)	502 (28.6)	
Yes	4699 (68.1)	3446 (66.9)	1253 (71.4)	
Arthriti, n (%)				< 0.001
No	4577 (69.5)	3592 (72.5)	985 (60.3)	
Yes	2012 (30.5)	1364 (27.5)	648 (39.7)	
Obese, n (%)				< 0.001
No	4115 (59.7)	3190 (62.1)	925 (52.7)	
Yes	2774 (40.3)	1945 (37.9)	829 (47.3)	
CVD, n (%)				< 0.001
No	5832 (88.4)	4432 (89.2)	1400 (85.7)	
Yes	768 (11.6)	534 (10.8)	234 (14.3)	
COPD, n (%)				< 0.001
No	6397 (96.9)	4861 (97.9)	1536 (94)	
Yes	203 ( 3.1)	105 (2.1)	98 (6)	
CKD, n (%)				0.008
No	5307 (80.6)	4009 (81.3)	1298 (78.3)	
Yes	1281 (19.4)	922 (18.7)	359 (21.7)	
Asthma, n (%)				< 0.001
No	5955 (86.3)	4536 (88.1)	1419 (80.9)	
Yes	948 (13.7)	612 (11.9)	336 (19.1)	
Stroke, n (%)				< 0.001
No	6314 (95.9)	4788 (96.7)	1526 (93.4)	
Yes	268 ( 4.1)	161 (3.3)	107 (6.6)	
Hypertension, n (%)				0.041
No	3959 (57.4)	2916 (56.6)	1043 (59.4)	
Yes	2944 (42.6)	2232 (43.4)	712 (40.6)	
Hyperlipidemia, n (%)				0.826
No	2193 (31.8)	1632 (31.7)	561 (32)	
Yes	4709 (68.2)	3516 (68.3)	1193 (68)	
Diabetes mellitus, n (%)				0.238
No	5623 (81.5)	4210 (81.8)	1413 (80.5)	
Yes	1280 (18.5)	938 (18.2)	342 (19.5)	
Cancer, n (%)				< 0.001
No	5723 (86.7)	4253 (85.6)	1470 (90)	
Yes	877 (13.3)	713 (14.4)	164 (10)	

### Multivariate regression analysis

In a multivariate regression model including [gender, age,race, smoke, alcohol, multimorbidity], a higher theobromine intake was associated with increased risk of depression (OR:1.17, 95%CI:1.02–1.34, AIC 319.528; Table 2). A subgroup analysis revealed that in participants aged < 60 years [*p *< 0.001], without multimorbidity [*p* < 0.001], obesity [*p* < 0.05] or cancer [*p* = 0.002], higher theobromine intake was associated with increased risk for depression (Table 3).

**Table 2:** Association of theobromine with depression

Variables	OR [95%CI]	*P*-value
**Theobromine intake**		
< 43 mg/day	1(Ref)	
> = 43 mg/day	1.17 (1.02 ~ 1.34)	0.023
**Age**	0.98 (0.98 ~ 0.98)	< 0.001
**Sex**		
Female	1(Ref)	
Male	0.79 (0.69 ~ 0.9)	0.001
**Smoke**		
Former	1(Ref)	
Never	0.55 (0.46 ~ 0.65)	< 0.001
Now	0.92 (0.75 ~ 1.13)	0.423
**Alcohol user**		
Heavy	1(Ref)	
Mild	0.69 (0.58 ~ 0.83)	< 0.001
Moderate	0.74 (0.6 ~ 0.91)	0.004
Never	0.48 (0.37 ~ 0.62)	< 0.001
**Multimorbidity**		
No	1(Ref)	
Yes	1.65 (1.41 ~ 1.93)	< 0.001
**Race**		
Black	1(Ref)	
Mexican American	0.83 (0.62 ~ 1.12)	0.224
White	1.53 (1.26 ~ 1.85)	< 0.001
Other Race	1.09 (0.88 ~ 1.35)	0.429

**Table 3:** Subgroup analyses

Subgroup	Theobromine intake	N total	N event_%	OR_95CI	*P* value	P for interaction
Age: < 40 year						0.001
	< 43 mg/day	1300	352 (27.1)	1(Ref)		
	> = 43 mg/day	755	263 (34.8)	1.42 (1.17 ~ 1.72)	< 0.001	
Age: 40-60 year						
	< 43 mg/day	1527	323 (21.2)	1(Ref)		
	> = 43 mg/day	689	175 (25.4)	1.31 (1.06 ~ 1.63)	0.012	
Age: > 60 year						
	< 43 mg/day	1850	467 (25.2)	1(Ref)		
	> = 43 mg/day	782	175 (22.4)	0.86 (0.71 ~ 1.06)	0.153	
Female						0.145
	< 43 mg/day	2528	682 (27)	1(Ref)		
	> = 43 mg/day	1035	338 (32.7)	1.29 (1.11 ~ 1.51)	0.001	
Male						
	< 43 mg/day	2149	460 (21.4)	1(Ref)		
	> = 43 mg/day	1191	275 (23.1)	1.07 (0.91 ~ 1.27)	0.408	
PIR: high						0.535
	< 43 mg/day	1854	250 (13.5)	1(Ref)		
	> = 43 mg/day	780	109 (14)	1.05 (0.82 ~ 1.34)	0.684	
PIR:low						
	< 43 mg/day	947	362 (38.2)	1(Ref)		
	> = 43 mg/day	550	236 (42.9)	1.14 (0.92 ~ 1.42)	0.235	
PIR:medium						
	< 43 mg/day	1400	441 (31.5)	1(Ref)		
	> = 43 mg/day	621	199 (32)	1.05 (0.86 ~ 1.29)	0.633	
Education: Below high school						0.903
	< 43 mg/day	509	160 (31.4)	1(Ref)		
	> = 43 mg/day	315	104 (33)	1.06 (0.78 ~ 1.43)	0.707	
Education:High school						
	< 43 mg/day	1015	320 (31.5)	1(Ref)		
	> = 43 mg/day	578	203 (35.1)	1.11 (0.89 ~ 1.38)	0.343	
Education: College or above						
	< 43 mg/day	3151	661 (21)	1(Ref)		
	> = 43 mg/day	1329	306 (23)	1.12 (0.96 ~ 1.31)	0.15	
Smoke:former						0.219
	< 43 mg/day	1052	282 (26.8)	1(Ref)		
	> = 43 mg/day	518	162 (31.3)	1.24 (0.98 ~ 1.56)	0.074	
Smoke: never						
	< 43 mg/day	2836	536 (18.9)	1(Ref)		
	> = 43 mg/day	1241	263 (21.2)	1.11 (0.94 ~ 1.31)	0.212	
Smoke: now						
	< 43 mg/day	789	324 (41.1)	1(Ref)		
	> = 43 mg/day	467	188 (40.3)	0.97 (0.77 ~ 1.23)	0.83	
Alcohol.user: heavy						0.522
	< 43 mg/day	641	216 (33.7)	1(Ref)		
	> = 43 mg/day	397	150 (37.8)	1.14 (0.87 ~ 1.48)	0.342	
Alcohol.user: mild						
	< 43 mg/day	1736	376 (21.7)	1(Ref)		
	> = 43 mg/day	829	195 (23.5)	1.1 (0.9 ~ 1.34)	0.35	
Alcohol.user: moderate						
	< 43 mg/day	718	170 (23.7)	1(Ref)		
	> = 43 mg/day	287	87 (30.3)	1.4 (1.04 ~ 1.91)	0.029	
Alcohol user: never						
	< 43 mg/day	539	70 (13)	1(Ref)		
	> = 43 mg/day	244	42 (17.2)	1.39 (0.91 ~ 2.1)	0.124	
Multimorbidity: no						0.008
	< 43 mg/day	1435	290 (20.2)	1(Ref)		
	> = 43 mg/day	769	212 (27.6)	1.45 (1.18 ~ 1.78)	< 0.001	
Multimorbidity: yes						
	< 43 mg/day	3242	852 (26.3)	1(Ref)		
	> = 43 mg/day	1457	401 (27.5)	1.04 (0.91 ~ 1.2)	0.538	
Arthriti: no						0.067
	< 43 mg/day	3109	628 (20.2)	1(Ref)		
	> = 43 mg/day	1468	357 (24.3)	1.25 (1.08 ~ 1.46)	0.003	
Arthriti: yes						
	< 43 mg/day	1381	444 (32.2)	1(Ref)		
	> = 43 mg/day	631	204 (32.3)	0.94 (0.77 ~ 1.16)	0.581	
Obese: no						< 0.001
	< 43 mg/day	2749	557 (20.3)	1(Ref)		
	> = 43 mg/day	1366	368 (26.9)	1.42 (1.22 ~ 1.65)	< 0.001	
Obese: yes						
	< 43 mg/day	1922	584 (30.4)	1(Ref)		
	> = 43 mg/day	852	245 (28.8)	0.91 (0.76 ~ 1.09)	0.32	
CVD:no						0.084
	< 43 mg/day	3935	897 (22.8)	1(Ref)		
	> = 43 mg/day	1897	503 (26.5)	1.21 (1.07 ~ 1.38)	0.003	
CVD: yes						
	< 43 mg/day	566	176 (31.1)	1(Ref)		
	> = 43 mg/day	202	58 (28.7)	0.79 (0.55 ~ 1.14)	0.208	
COPD:no						0.994
	< 43 mg/day	4378	1015 (23.2)	1(Ref)		
	> = 43 mg/day	2019	521 (25.8)	1.14 (1.01 ~ 1.28)	0.04	
COPD: yes						
	< 43 mg/day	123	58 (47.2)	1(Ref)		
	> = 43 mg/day	80	40 (50)	1.37 (0.74 ~ 2.54)	0.309	
CKD: no						0.157
	< 43 mg/day	3563	842 (23.6)	1(Ref)		
	> = 43 mg/day	1744	456 (26.1)	1.12 (0.98 ~ 1.28)	0.092	
CKD:yes						
	< 43 mg/day	910	238 (26.2)	1(Ref)		
	> = 43 mg/day	371	121 (32.6)	1.39 (1.06 ~ 1.81)	0.015	
Asthma: no						0.953
	< 43 mg/day	4051	928 (22.9)	1(Ref)		
	> = 43 mg/day	1904	491 (25.8)	1.14 (1 ~ 1.29)	0.047	
Asthma: yes						
	< 43 mg/day	626	214 (34.2)	1(Ref)		
	> = 43 mg/day	322	122 (37.9)	1.21 (0.91 ~ 1.61)	0.18	
Stroke: no						0.002
	< 43 mg/day	4291	985 (23)	1(Ref)		
	> = 43 mg/day	2023	541 (26.7)	1.21 (1.07 ~ 1.37)	0.002	
Stroke: yes						
	< 43 mg/day	196	87 (44.4)	1(Ref)		
	> = 43 mg/day	72	20 (27.8)	0.45 (0.25 ~ 0.82)	0.009	
Hypertension: no						0.557
	< 43 mg/day	2646	672 (25.4)	1(Ref)		
	> = 43 mg/day	1313	371 (28.3)	1.14 (0.98 ~ 1.32)	0.087	
Hypertension:yes						
	< 43 mg/day	2031	470 (23.1)	1(Ref)		
	> = 43 mg/day	913	242 (26.5)	1.21 (1.01 ~ 1.45)	0.04	
Hyperlipidemia: no						0.26
	< 43 mg/day	1440	362 (25.1)	1(Ref)		
	> = 43 mg/day	753	199 (26.4)	1.04 (0.85 ~ 1.28)	0.672	
Hyperlipidemia: yes						
	< 43 mg/day	3237	780 (24.1)	1(Ref)		
	> = 43 mg/day	1472	413 (28.1)	1.21 (1.05 ~ 1.39)	0.008	
DM: no						0.646
	< 43 mg/day	3773	904 (24)	1(Ref)		
	> = 43 mg/day	1850	509 (27.5)	1.17 (1.03 ~ 1.33)	0.017	
DM: yes						
	< 43 mg/day	904	238 (26.3)	1(Ref)		
	> = 43 mg/day	376	104 (27.7)	1.07 (0.82 ~ 1.41)	0.605	
Cancer: no						0.029
	< 43 mg/day	3901	954 (24.5)	1(Ref)		
	> = 43 mg/day	1822	516 (28.3)	1.22 (1.08 ~ 1.39)	0.002	
Cancer: yes						
	< 43 mg/day	600	119 (19.8)	1(Ref)		
	> = 43 mg/day	277	45 (16.2)	0.73 (0.49 ~ 1.09)	0.125	

### Relationship between theobromine and depression

**Fig. 2:**
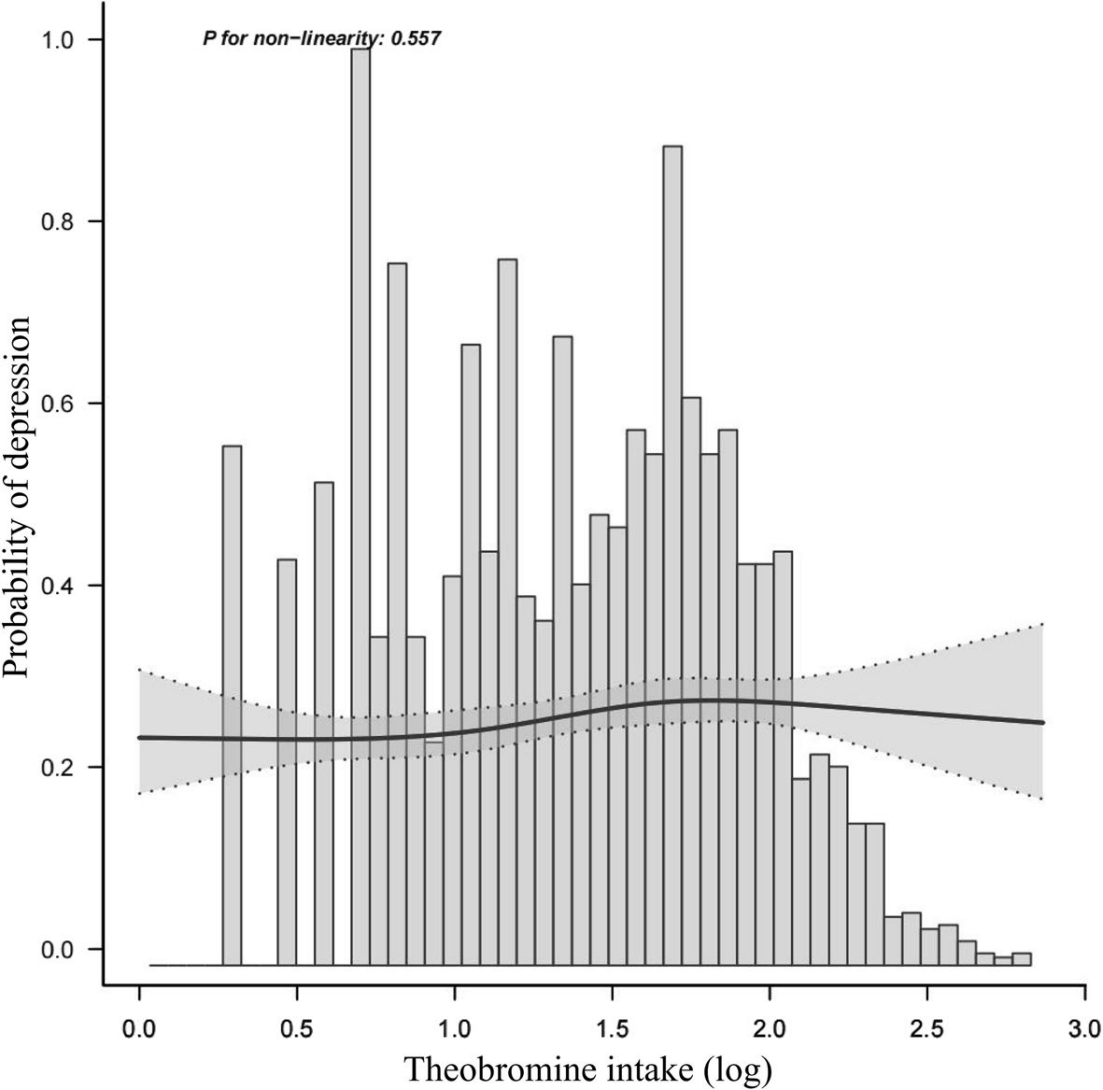
Dose–response relationship between theobromine and depression

A restricted cubic spline (RCS) was used to furtherly clarify the relationship between theobromine and depression after controlling for possible variables. Figure 2 suggests that theobromine is positively correlated with the prevalence of depression.

## Discussion

In this study, we identified an association between increased theobromine consumption and depressive symptoms, even after adjusting for age, sex, race, multimorbidity, smoking status, and alcohol consumption, these relationships were still clearly visible.

Our findings are in the line of previous studies reporting protective effects of theobromine on cognitive function. A wide range of mechanisms have been suggested in the literature such as improved neurotransmission, upregulation of brain derived neurothrophic factors and modulation of calcium and phosphodiesterase homeostasis [22]. Furthermore, experimental findings showed how theobromine is able to cross the blood brain barrier where it regulates the activity of neurotransmitter receptors, such as adenosine receptors, which have been linked to depressive and anxiety states [23, 24]. Other adenosine receptor independent effects were reported such as the reduction of cellular oxidative stress and upregulation of gene expression [PRDX1、PRDX6] [24].

Our findings contrast with previous studies reporting that increased consumption of caffeine, which is also a methylxanthine, is associated with decreased risk of depression [23, 25, 26]. Indeed, a study conducted in the United Kingdom found that unemployed individuals consuming caffeine on a regular basis were more likely to report depresssive symptoms [27]. Although belonging to the same group, pharmacological differences are noted between caffeine and theobromine and may therefore explain the opposite effects on mood but also on blood pressure [24, 25]. Caffeine is metabolised to theobromine in the liver and studies conducted in the rat and in humans, show that theobromine has a higher half-life than caffeine, which is more rapidly degraded [24, 28]. Hence, it is believed that the beneficial effect of caffeine is mediated through its metabolites, such as theobromine [24, 25]. It is also hypothesised that the effects of caffeine are more CNS specific, resulting in symptoms such as alertness, while theobromine exerts its effect primarily via peripheral changes [25]. However, the differences between the two compounds, and their effect on mood stated need to be further explored.

Our subgroup analysis shows that younger participants (i.e. under 60 years old) were more likely to report depression with increased theobromine intake. Previous studies have suggested that young age during pregnancy [especially below 26 years of age] are at increased risk for anxiety and depression [29]. But the pharmacological properties of theobromine may also be affected by recall bias in the elderly, which could not be completely excluded from the questionnaire, and by the effects of oral administration of multiple drugs in the elderly population. Also, our study showed a positive association between theobromine and depressive symptoms in participants without multimorbidity. By showing an association between theobromine consumption and depression, our study further fuels the debate on the role of nutrition in mental health care and particularly in risk groups. For example, previous studies have found polyunsaturated fatty acids (PUFAs), which may affect depression in elderly japanese people [30].

However, some limitations remain. Due to the inability of cross-sectional observational studies to establish causality and directionality, our results should be regarded with caution. In addition, the effect of caffeine could not be investigated due to the data paucity. Furthermore, despite thorough adjustments for confounding, residual confounding cannot be ruled out. In particular, recall bias from older adults cannot be completely excluded.

## Conclusion

Our study suggests that theobromine intake is associated with increased risk for depression in adults, highlighting the importance of nutrition on the cognitive function. Finally, further studies are needed to clarify the link between theobromine and mood states.

## Data Availability

The National Health and Nutrition Examination Survey (NHANES) data are publically available at https:// wwwn.cdc.gov/nchs/nhanes which is publicly available. Accession number: NHANES 2017–2018.
